# Editorial: Molecular mechanisms of neuropsychiatric diseases, volume II

**DOI:** 10.3389/fnmol.2024.1517196

**Published:** 2024-11-13

**Authors:** Mónica Moreira-Rodrigues

**Affiliations:** Department of Immuno-Physiology and Pharmacology, Laboratory of General Physiology and Center for Drug Discovery and Innovative Medicines (MedInUP), School of Medicine and Biomedical Sciences (ICBAS), University of Porto (UP), Porto, Portugal

**Keywords:** neuropsychiatric diseases, molecular mechanisms, therapeutic strategies, post-traumatic stress disorder, catecholamines, epinephrine, dopamine, dopamine β-hydroxylase inhibitor

Neuropsychiatric disorders affect millions of people globally. Despite their high prevalence, the precise molecular mechanisms behind these conditions remain largely unclear. A complex interplay of genetic, developmental, and environmental factors may increase susceptibility to these disorders, with specific molecular changes potentially influencing disease onset and progression. Understanding these molecular pathways is crucial for deepening our knowledge of neuropsychiatric pathophysiology and for developing targeted therapies. The Research Topic Molecular Mechanisms of Neuropsychiatric Diseases is the second volume and the continuation of the first volume (Moreira-Rodrigues and Grubisha, 2022), being created in response to the high interest and positive reception it received in the first volume.

This Research Topic aims to uncover key molecular mechanisms in neuropsychiatric disorders and explore how they might inform therapeutic strategies. The progress in understanding these disorders has been slowed by a lack of clear anatomical and molecular mechanism understanding. The papers in this Research Topic address these obstacles by connecting signs and symptoms to underlying anatomical and molecular alterations.

The review by Azevedo et al. examines how the activation of the sympathoadrenal medullary system and the hypothalamic-pituitary-adrenal axis in response to stress impacts memory formation, particularly in fear and traumatic memories linked to post-traumatic stress disorder (PTSD). Catecholamines like norepinephrine (NE) and epinephrine (EPI) play key roles in adaptive responses to stress and are involved in contextual fear memory enhancement through β_2_-adrenoceptor (β_2−_AR) activation (Alves et al., 2016), influencing the brain via the vagus nerve and blood glucose changes (Oliveira et al., 2024, 2023). This review highlights that EPI is particularly influential in fear memories, activating the hippocampal Nr4a2 mRNA expression related to cAMP response element-binding protein (CREB), which plays a significant role in contextual fear memory consolidation under stressful conditions (Oliveira et al., 2018). An imbalance in this system, especially over-active sympathetic responses, contributes to the persistence of traumatic memories in PTSD, with some patients (Pan et al., 2018) and animal PTSD models showing elevated catecholamine levels (Martinho et al., 2020). The authors suggest that treatments such as sotalol, a β-AR blocker (Martinho et al., 2021a), and nepicastat, a dopamine β-hydroxylase inhibitor (Martinho et al., 2021b), could reduce PTSD risk by preventing the consolidation of traumatic memories. These drugs appear to regulate gene expression in the hippocampus (Nr4a, Npas4, or Bdnf), potentially fostering more neutral contextual memories (Martinho et al., 2021a,b). These treatment approaches, combined with therapy, may help PTSD patients build resilience and cope with trauma by down-regulating excessive catecholamine effects and dysregulated gene activity.

A brief research report article by Bartsch et al. investigates how traumatic stress leads to persistent neural changes, focusing on synaptic plasticity in a pathway between the neurons of the posterior ventral segment of the medial amygdala and the ventrolateral segment of the ventromedial hypothalamus. Using the mGRASP imaging technique, researchers observed an increase in synapse formation in this pathway in mice exposed to acute stress compared to controls. Chemogenetic inhibition of CaMKIIα-expressing neurons in the medial amygdala during stress reduced these synaptic changes, suggesting excitatory activity drives this plasticity. Additionally, blocking NMDA receptors prevented stress-induced synapse formation, highlighting an NMDA receptor-dependent mechanism. These structural changes in the medial amygdala and the ventromedial hypothalamus pathway may underlie lasting behavioral effects of traumatic stress, such as those seen in PTSD and social behavior changes, shedding light on potential targets for intervention.

A brief research report article by Petri et al. examines how dopamine transporter (DAT) deficiency affects stress responses, highlighting the role of dopaminergic neurotransmission in stress susceptibility and resilience. Researchers compared wild-type (WT) and DAT-knockout (KO) mice under chronic stress, observing that DAT-KO mice showed resistance to stress-related anxiety behaviors. These findings suggest that loss of DAT may protect against stress-induced anxiety-like behaviors, underscoring the significance of dopamine pathways in stress resilience.

The original research article of Bielopolski et al. explored how specific mutations in the GABBR2 gene, which encodes a subunit of the GABA_B_ receptor (GABA_B_R) critical for inhibitory neurotransmission in the brain, impact receptor structure and function. A 7-year-old boy with Level 3 Autism Spectrum Disorder was found to carry a *de novo* GABBR2 p.Arg212Gln variant, identified as a variant of unknown significance via whole exome sequencing. Although treatment with the GABA_B_R agonist baclofen showed no improvement, researchers investigated whether this variant might contribute to patient‘s symptoms. Using molecular dynamics simulations and *in vitro* experiments, they studied how the p.Arg212Gln variant, along with the previously identified p.Arg212Trp variant, affects GABA_B_R. Both variants alter the receptor's extracellular domain and affect the transmembrane helices. p.Arg212Gln shifts the receptor toward an active state, increasing constitutive activity and GABA potency, while p.Arg212Trp shifts it toward an inactive state, reducing GABA activity. These findings highlight the potential of molecular dynamics simulations in evaluating variants of unknown significance, which may guide personalized treatments by clarifying variant-specific receptor associated behavior.

In conclusion, this Research Topic showcases innovative approaches that deepen our understanding of the molecular mechanisms underlying neuropsychiatric disorders. By integrating anatomical, cellular, molecular, and genetic findings, these studies highlight potential therapeutic strategies, such as targeting catecholamine dysregulation to help PTSD patients build resilience and cope with trauma. Insights into structural changes in pathways between the medial amygdala and ventromedial hypothalamus further reveal how these alterations may drive persistent stress-related behaviors, offering new avenues for intervention. Additionally, findings on dopamine transporter deficiency suggest protective effects against stress-induced anxiety, emphasizing dopamine's role in resilience. Finally, the use of molecular dynamics simulations illustrates how assessing variants of unknown significance can clarify receptor-specific behavior and inform personalized treatments. Altogether, this Research Topic of studies paves the way toward the identification of novel therapeutic targets and rational drug design in the field.
